# Knocking on Closed Doors: Host Interferons Dynamically Regulate Blood-Brain Barrier Function during Viral Infections of the Central Nervous System

**DOI:** 10.1371/journal.ppat.1005096

**Published:** 2015-09-17

**Authors:** Brian P. Daniels, Robyn S. Klein

**Affiliations:** 1 Department of Anatomy and Neurobiology, Washington University School of Medicine, St. Louis, Missouri, United States of America; 2 Department of Internal Medicine, Washington University School of Medicine, St. Louis, Missouri, United States of America; 3 Department of Pathology and Immunology, Washington University School of Medicine, St. Louis, Missouri, United States of America; University of Kentucky, UNITED STATES

## Introduction

The central nervous system (CNS) is among the most important organ systems, integrating information inputs and coordinating the activity of all other body systems. Like many organ systems, the CNS is susceptible to infection by pathogenic microorganisms, including many arboviruses that are considered neurotropic because they are able to achieve robust replication in neural cells. Neurotropic arboviruses capable of infecting the CNS include members of the Flaviviridae (e.g., West Nile and Japanese encephalitis viruses), Bunyaviridae (La Cross and Rift Valley Fever viruses), and Togaviridae (Alphavirus species) families, all RNA viruses that are maintained in complex life cycles involving a nonhuman primary vertebrate and a primary arthropod vector [1]. A variety of mechanisms exist to protect the CNS from the entry and infection of neurotropic viruses, including innate immune responses and multilayer barriers formed by diverse host cell types [2]. However, many arboviruses gain access to the CNS either as free virions, within motile infected cells, and/or by using axonal transport mechanisms of peripheral nerves that directly enter or form synapses within the CNS. Viruses that enter via the bloodstream must cross CNS endothelial barriers that exhibit unique specializations, collectively termed the blood-brain barrier (BBB).

## The Blood-Brain Barrier

The CNS is normally protected from pathogens in the circulation by the BBB. The BBB is a dynamic interface that limits the passage of molecules and cells from the blood to the brain, protecting neural cells from injury [3,4]. It is formed by highly specialized brain microvascular endothelial cells (BMECs) joined by tight (TJ) and adherens junctions (AJ) with associated pericytes and enveloped by astrocytic endfeet [5]. These TJ and AJ complexes effectively seal the paracellular space between BMECs, preventing the movement of pathogens and pathogen-infected cells in the blood into the CNS parenchyma [6]. Disruption of the BBB is a hallmark of CNS infections and can be caused by both viral factors and the host immune response [2]. Recent research has highlighted how a major family of antiviral cytokines, the interferons (IFNs), plays multifaceted roles at the BBB during neurotropic viral infections. This review will summarize recent investigations that have expanded our understanding of how host IFNs serve to protect the CNS during viral infections.

## Type I IFN

The type I IFNs consist of the ligands IFN-β and 13 IFN-α subtypes, which each signal through a common IFN-α receptor (IFNAR) that is expressed by nearly all nucleated cells in the body. Type I IFNs are rapidly induced during viral infection by host detection of pathogen-associated molecular patterns (PAMPs), and their role in restricting viral pathogenesis has been extensively characterized [7]. These functions include induction of the antiviral state in both infected and bystander host cells. Type I IFN signaling via the canonical JAK/STAT pathway leads to the expression of a panoply of interferon stimulated genes (ISGs), many of which are absolutely essential for restriction of viral infections and effective viral clearance [7,8]. In addition to the traditional antiviral functions of type I IFNs, however, a growing body of research has established critical functions for type I IFN at the BBB during inflammatory diseases of the CNS.

The first indications that type I IFNs could modulate BBB function were discovered in the context of CNS autoimmunity. In animal models and in vitro BBB cultures consisting of BMECs and astrocytes grown in transwell systems, treatment with type I IFNs decreases BBB permeability, enhances TJ integrity, and limits the migration of leukocytes across the BBB into the CNS parenchyma [9–11], effects that contribute to the efficacy of IFN-β as a treatment for the CNS autoimmune disease multiple sclerosis. However, although type I IFNs have been known to preserve BBB integrity in the context of autoimmunity for some time, the potential effects of these antiviral cytokines on the BBB during neurotropic viral infections have only recently been addressed.

Indeed, recent work has established a novel antiviral function for type I IFNs at the BBB. The induction of type I IFN expression following detection of viral pathogens such as West Nile virus (WNV) acts directly on BBB endothelium to preserve TJ formation and limit BBB permeability [11]. This effect is mediated by preferential activation of the cytoskeletal regulatory GTPase Rac1, which is known to enhance endothelial barrier function. This effect prevents and/or reverses the activation of the opposing GTPase RhoA, whose activation downstream of inflammatory signals leads to loss of TJ integrity and barrier function [11,12]. In addition, type I IFNs act indirectly to preserve BBB integrity by limiting the expression of barrier-disrupting inflammatory cytokines, including TNF-α and IL-1β [8,11]. While the relative contributions of circulating serum IFNs versus local CNS IFN expression to these processes remains unclear, it is likley that IFN signalling on both sides of the BBB works to preserve barrier integrity during neurotropic viral infection [2,11].

## Type II IFN

The type II IFN family consists solely of IFN-γ, an inflammatory cytokine that signals through the IFN-γ receptor (IFNGR). In contrast to type I IFNs, type II IFN is most associated with adaptive immune responses. The secretion of IFN-γ by Natural Killer (NK) cells and activated T cells is a major signal for the recruitment and activation of leukocytes to sites of infection. While the actions of IFN-γ at the BBB have often been considered to be secondary consequences of its role in leukocyte recruitment, vascular endothelia can also respond directly to IFN-γ stimulation. Addition of IFN-γ to vascular endothelia in vitro dysregulates barrier function, leading to enhanced permeability [11]. Recent research has identified IFN-γ as a driver of BBB permeability in the context of CNS infections, including pneumococcal meningitis [13] and rabies virus (RabV) infection [14]. Putative mechanisms for direct BBB dysregulation by IFN-γ include the down-regulation and/or internalization of TJ proteins [14,15].

In addition to direct effects on BBB endothelium, the enhancement of leukocyte trafficking and activation by IFN-γ also serves to disrupt BBB function by promoting the expression of other inflammatory cytokines and chemokines. Most notably, IFN-γ expression in response to CNS infections is a potent inducer of the lymphocyte chemoattractant CXCL10. IFN-γ-mediated stimulation of CXCL10 expression enhances the recruitment of yet more IFN-γ–expressing T-lymphocytes to sites of infection, establishing a feedforward mechanism by which increasing numbers of inflammatory cells traffic to sites of infection and produce inflammatory mediators that perturb BBB function. This IFN-γ/CXCL10 axis is a major source of BBB breakdown and neuroinflammation during several CNS infections, including those caused by RabV [14] and Human T-lymphotropic virus-1 (HTLV-1) [16]. While the disruption of the BBB due to inflammation can be a route of access for circulating pathogens and a source of tissue injury, it is often also necessary for effective cell-mediated immunity in the CNS and complete pathogen clearance [2].

## Type III IFN

The recently classified type III IFNs are composed of three IFN-λ subtypes, each of which signal through a receptor complex made up of the IFN-λ receptor (IFNLR1) and an IL10Rβ subunit. Though, like type I IFNs, they are induced by host detection of PAMPs, the expression of type III IFNs is primarily restricted to tissue barriers, including intestinal, airway, vaginal, and skin epithelia [17]. Though IFN-λ signals through similar mechanisms as type I IFNs, recent research has demonstrated that IFN-λ plays essential, nonredundant antiviral functions in barrier epithelia in the context of gastrointestinal infections [18–20]. However, though the roles of IFN-λ at epithelial barriers during viral infections have been increasingly well characterized, it has remained unclear whether IFN-λ also signals at endothelial barriers.

In a recent study by our group and others, we showed that IFNLR1 is expressed on neurovascular cells, including BMECs and astrocytes [21]. In the setting of WNV infection, IFN-λ signalling at the BBB served to preserve BBB function by enhancing TJ integrity, thereby limiting the neuroinvasive potential of WNV. This effect was observed in the absence of direct antiviral effects of IFN-λ on neurons or other cells normally targeted by WNV. IFN-λ-mediated effects on BBB function occur through poorly understood mechanisms that are independent of protein synthesis and STAT1 activation. In addition, *Ifnlr1* ^*-/-*^ exhibited normal adaptive immune responses following WNV infection, in contrast to mice deficient in type I IFN signalling [8]. Thus, though IFN-λ preserves BBB integrity through similar mechanisms as type I IFN during WNV infection, the restriction of IFNLR1 to tissue barriers and the specificity of IFN-λ signaling to effects on the BBB during WNV infection make IFN-λ an exciting potential therepeutic option for neuroinvasive infections and other diseases that involve breakdown of the BBB, including CNS autoimmunity.

## Concluding Remarks

**Fig 1:**
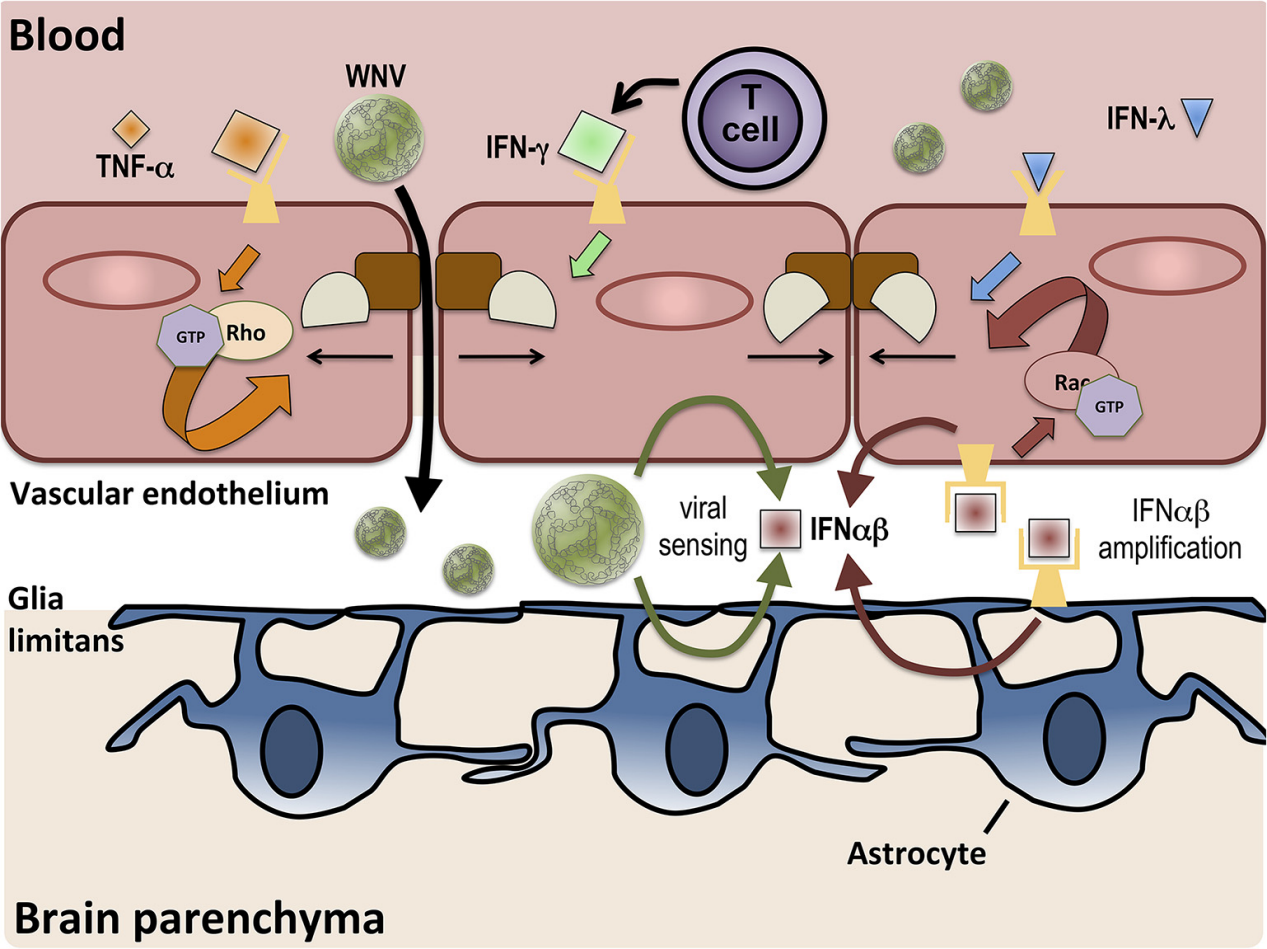
BBB regulation occurs via signaling on both lumenal and ablumenal sides of CNS vascular endothelium. Serum cytokines, such as TNF-α (orange diamonds), induce tight junction (brown and tan shapes) disruption via receptor-mediated activation (orange arrows) of RhoA GTPases. T cell-derived IFN-γ (green diamond) also increases BBB permeability. BBB crossing of WNV leads to increased expression of IFNαβ (red square) via viral sensing pathways (thin green arrows). IFNαβ expression is amplified (thin red arrows) in both endothelial cells and astrocytes and enhances tight junction integrity via activation of Rac1 (red arrows). IFN-λ (blue triangle) signaling also closes the BBB, preventing the entry of virus.

Viral infections of the CNS remain an important cause of morbidity and mortality worldwide, primarily due to the effects of virus invasion and/or to pathologic CNS entry of immune cells. Here we have highlighted some of the distinct signaling pathways whereby interferons may regulate virus versus immune cell entry into the CNS at the BBB at lumenal and abluminal surfaces (see Fig 1). The separation of these processes appears to be an adaptive response that allows the CNS to protect itself from infection with virus while, at the same time, promoting viral clearance. It is clear that the elucidation of factors and pathways that control these mechanisms is essential for the development of strategies to limit CNS infections while promoting normal immune function. However, the potential for coincidental signaling of these pathways in certain individuals may ultimately provide the explanation for how viruses may modulate barrier function such that immune privilege is compromised, triggering CNS autoimmunity. Indeed, the interactions between type II IFN–expressing immune cells with type I IFN–expressing BMECs suggest that opposing signals may converge on the BBB. The kinetics of this process may determine whether the BBB remains closed or opens, as the presence of virus at the BBB may be temporary. Further studies are needed to define essential mechanisms of innate immunity at the BBB, the impact of viral infections on converging signaling pathways, and their links to neuropathologic processes.
